# A qualitative study exploring the benefits of involving young people in mental health research

**DOI:** 10.1111/hex.13722

**Published:** 2023-04-19

**Authors:** Rebecca Watson, Lowrie Burgess, Elise Sellars, Jodie Crooks, Rose McGowan, James Diffey, Georgia Naughton, Rebekah Carrington, Cassie Lovelock, Rachel Temple, Cathy Creswell, Christina McMellon

**Affiliations:** ^1^ Departments of Experimental Psychology and Psychiatry University of Oxford Oxford UK; ^2^ Young People's Network The McPin Foundation London UK; ^3^ Social and Public Health Sciences Unit University of Glasgow Glasgow UK

**Keywords:** co‐researchers, mental health, PPI, young people

## Abstract

**Introduction:**

It is increasingly accepted that young people need to be centrally involved in research on issues that affect them. The aim of this study was to explore young people's perceptions of the benefits for them of being involved in mental health research and the processes that enabled these benefits.

**Methods:**

Qualitative interviews were conducted by co‐researchers (young people with lived experience and/or interest in mental health) with 13 young people (aged 13–24 years) who had experience of being involved in mental health research when they were between 11 and 16 years of age. Reflective thematic analysis was used to identify important aspects of young people's experiences.

**Results:**

Four main themes were identified: (1) opportunity to have a meaningful impact, (2) opportunity to be part of a supportive community, (3) opportunity to learn and grow and (4) increasing opportunities for young people.

**Conclusion:**

This study highlights young people's experiences of being involved in mental health research and identifies ways in which researchers can ensure that involvement opportunities bring benefits to both the young people and the research.

**Patient or Public Contribution:**

This research was a response to issues raised by young people involved in research. The project was supported by co‐researchers throughout, including design, data collection, analysis and write‐up.

## 1 INTRODUCTION

Recent data from the NHS indicated that one in six children aged 5–16 years in England had a likely mental health difficulty in 2020.^1^ Experiencing mental health difficulties during adolescence is associated with a range of poor health and educational outcomes.^2^ There is a clear consensus that more research is needed to understand how best to prevent and address the high mental health need among adolescents.^3^ Furthermore, it is increasingly accepted that young people need to be centrally involved in that research.^4^

This push for greater involvement of young people in mental health research builds on research that demonstrates that studies that involve people with lived experience are more robust, more likely to achieve target recruitment and enhance the translation of findings into practice.^5^ Therefore, involving young people in research may improve a study's acceptability and impact.^6^ It is also young people's right to be involved in decision‐making about issues that affect them, in line with the 1989 United Nations Convention on the Rights of the Child.^7^ This paper explores a third key reason for involving young people in research processes, which is the benefits that young people see for being involved in mental health research.

The National Institute of Health Research (NIHR) defines Patient and Public Involvement (PPI) as research ‘carried out “with” or “by” members of the public rather than “to,” “about” or “for” them’.^8^ Different levels of collaboration between young people and researchers can all be described as ‘involvement’; to illustrate, young people's involvement has been used to describe light consultation or affirmation of researchers' decisions by young people but has also been used to describe research that is entirely youth‐led or co‐produced.^9^ Young people's ‘research involvement’, therefore, captures a variety of experiences, including advising for, collaborating on and leading research about young people. The concepts of PPI and involvement in research have been problematized by several researchers who point to the power imbalances that continue to exist in most research that involves young people and other stakeholders who are ‘invited in’ to academic research.^10^, ^11^ We recognize these challenges and note that the research and experiences presented in this paper originated in the university and that the benefits for young people do not negate these challenges.

Involving young people in research can be a way for young people to have their voices heard on issues affecting their own and their peer's lives, as well as providing an opportunity for personal and skills development.^12^ It can also bring challenges, for example, balancing giving a voice to youth with personal experience of mental health issues against the risk of exposing young people to potentially distressing mental health information.^4^ However, research guidelines have traditionally focused on increasing the benefits for the researcher, outlining practical considerations that enable young people's involvement, but view positive outcomes to young people as positive by‐products rather than a central aim.^9^ Furthermore, there has been a general lack of understanding from researchers about how to meaningfully involve young people with lived experience of mental health difficulties in research, which can result in poor experiences for both parties.^13^ Understanding the benefits and challenges of being involved in mental health research from a young person's perspective is critical for developing recommendations for professionals to ensure that they involve young people in mental health research in ways that bring benefits both for the research and for the young people themselves.

There is a growing literature examining the process of young people's involvement in individual projects but this is still not common. Sellars et al.^9^ found that fewer than 1% of relevant studies reported details of young people's involvement in mental health research. Even less is known about the benefits for young people who have been involved in research.^14^ A narrative review discussing the needs and challenges of young people's advisory groups (YPAGs) in Europe suggested that involvement provides potential to develop a variety of transferable skills (e.g., presentations, project management, negotiation and decision‐making) and to develop and extend their social skills and networks.^12^ Furthermore, a qualitative study with an opportunistic sample of eight young people aged 14–24 years reported that participation may allow them to have a say in decisions that affect their lives, to make an active contribution to their communities and to improve services used by children and young people.^14^ A picture is beginning to emerge of what and how young people can be involved in mental health research but there is still much to be learnt about what makes participation a meaningful experience for young people. This is particularly true for young people involved in mental health research between the ages of 11 and 16 years where parental consent is required and there may be developmental differences in the manner and extent of their involvement.

In this study, we explored young people's experiences of being involved in mental health research in response to issues raised by young advisors on other projects. We wanted to gain a better understanding of what makes involvement in mental health research enjoyable, useful or meaningful for young people as well as to understand any skills, knowledge and personal development opportunities that came from their involvement. We also wanted to give young people the chance to discuss their views on how young people should be involved in mental health research and how researchers can best support them.

## 2 METHOD

Guidelines for ensuring rigour and reflexivity in qualitative research were followed,^15^ as well as the COREQ checklist for reporting qualitative data.^16^

### 2.1 Co‐researchers

As well as investigating young people's experiences of being involved in mental health research, the present study also presents an example of co‐production, whereby young people, aged 18–24 years, with lived experience of mental health problems and experience of being involved in mental health research were employed as co‐researchers to work collaboratively with university researchers on data collection, analysis and reporting of results.^9^ Such involvement across the whole research process is viewed as central to the research design and builds on previous work with co‐researchers (see Fraser et al.^17^).

Researchers with experience conducting qualitative research (R. W., L. B., C. M.) lead training sessions with co‐researchers (J. C., J. D., R. M., R. C., G. N., C. L.) covering topics including a background to qualitative research, epistemology, reflexivity, rigour, memo writing, reflexive thematic analysis, code development and theme building. Peer‐to‐peer interviews were offered to participants as a way to reduce hierarchical relationships between researchers and interviewees, potentially enabling more honest discussions than could be had with an adult researcher.^18^ We worked collaboratively with the co‐researchers to interview young people and conduct a reflexive thematic analysis.

### 2.2 Participants

Thirteen young people aged between 13 and 24 years and based in the United Kingdom were interviewed. All of the participants had been involved in mental health research when they were between 11 and 16 years of age. The research in which they had been involved covered a range of mental health‐related topics (see Table 1) and some had additional experience in research beyond the topic of mental health. The 13 participants had taken part in at least 12 studies between them (many of the young people had taken part in multiple studies and could not always specify the exact number). Most participants were not known to each other; the maximum overlap was for four participants who had all taken part in two particular studies. Participants were sampled purposively for experience of involvement in different stages of mental health research, age, gender and ethnicity. Full demographic details can be found in Table 2. Names used within this paper are pseudonyms.

**Table 1:** Topics covered in participants' research experiences.

*Topics in mental health *
Young people's mental health (general) Impact of COVID‐19 on young people's mental health (general) CAMHS service improvement Creation of resources for young people's mental health after COVID‐19 Suicide Depression Eating disorders Anxiety Social disorder Mental health education for young people
*Other topics*
Voting age Online sexual abuse Sex education Climate change Gender equality Social inequality

**Table 2:** Participant characteristics.

Variable	Frequency
Gender	
Male	5
Female	9
Age	
11–13	1
14–16	6
17–19	1
20–24	5
Ethnicity	
White	7
Asian/Asian British	5
Black/Black British	1
Mixed	0
Chinese/Chinese British	0
Middle Eastern	0

### 2.3 Procedures

#### 2.3.1 Recruitment

Participants were recruited via an advert circulated to contacts in groups or studies known to have PPI, YPAGs or Young People's Networks. Young people aged 16 years and older were contacted directly and were sent a link to complete an online consent form. For young people younger than 16 years of age, contact was made via a parent/guardian, who was sent a link to complete an online consent form; once this was completed, the young person completed an online assent form. Thirteen young people were interviewed. Individuals who were not interviewed either did not respond to the email inviting them to arrange an interview or the interview could not be arranged to fit within their schedule.

#### 2.3.2 Interviews

Interviews were conducted online via Microsoft Teams. Participants indicated on the consent form whether they wanted to be interviewed by a researcher from the university research team; a trained co‐researcher; both or if they had no preference. Twelve participants indicated that they wanted to be interviewed by both or had no preference, and one participant opted to be interviewed by a researcher from the university research team only. Some participants were known to the researchers before they took part in their interview. These participants were involved in the YPAG, which was run by the researchers leading the study. Participants were made aware of who would be interviewing them before the interview, and had the choice to be interviewed by someone who they had no prior involvement with. Ahead of the interview, participants were provided with a short biography of the interviewer(s). At the beginning of each interview, the interviewer(s) explained that the purpose of the study was to learn about their experiences of taking part in mental health research when they were aged between 11 and 16 years. Participants older than 16 years of age and parents of participants younger than 16 years of age gave their verbal consent; participants younger than 16 years of age also provided their assent. Interviews were recorded using Microsoft Teams and an external audio‐recording device. Interviewers used a topic guide (see Supporting Information) developed based on the existing literature and in collaboration with co‐researchers to flexibly explore relevant aspects of participants' experiences. Following the interview, participants were emailed a £20 voucher to reimburse them for their time.

L. B. (female) met with the co‐researcher before each interview to give the co‐researcher relevant information about the participant (i.e., name, gender, age, involvement in research) and to answer any questions and to discuss how the co‐researcher was feeling before the interview. Co‐researchers (four female, one male) led the interview, with intermittent input from L. B. as cued by the co‐researcher. Following the interview, co‐researchers were debriefed by L. B. to check in on how they felt the interview went and address any worries that they may have had during the interview (e.g., about their interview technique; safeguarding concerns). Interviewers wrote reflective memos after each interview, considering their own sources of bias and prior assumptions, including knowledge and experience gained from personal or professional experience in the field of mental health. See Table 3 for reflections from co‐researchers on being involved in the research project.

**Table 3:** Co‐researchers' reflections on their experience of being a co‐researcher.

Area of reflection	Quotes from co‐researchers
Genuine collaboration and open communication	Working as a co‐researcher allowed me to use and improve on my research skills to work collaboratively with other members of the research team during the project. [There was an] acknowledgement that our experiences with mental health problems is a part of who we are and shapes our perspectives, but we have more to offer and contribute beyond those experiences and [we were] able to talk openly about previous/existing mental health problems without any pressure/expectations.
Flexibility	I think one thing that was done really well throughout the project is allowing co‐researchers flexibility surrounding their time commitments. Being understanding that often people have other commitments such as work/education that they often have to prioritise goes a long way. I think attempting to (as far as conveniently possible) use a time that suits everyone is great. Then, making sure everyone is aware that there's no pressure to do everything—just as much as they are able to.
Support and mentoring	There was a lot of support available for the co‐researchers during the project, I never felt isolated despite working from home. It was easy to get in touch with any of the member of staff involved with the project via email if we had any questions or needed help with anything at all, such as the lead researcher and research assistant and the head of PPI at McPin.
Practical constraints	[We were] restricted by hourly pay/only having a set number of funded hours on the project. [It is] hard to dip in and out of a project that requires this much thought, particularly with analysis—I often started my part of analysis, moved away from it for a little while to keep arranging my thoughts, then coming back and making cohesive points.
Training and wider opportunities	I think one of the most valuable parts of my involvement was the guidance from senior researchers. This was particularly helpful to me as an aspiring researcher. However, I think it may be beneficial more widely, too.

#### 2.3.3 Data analysis

Transcripts were generated using Microsoft Teams and edited for accuracy and clarity by L. B. Data were then managed in NVivo. We used Braun and Clarke's^19^, ^20^ reflexive thematic analysis approach to guide a process of collaborative analysis with the co‐researchers. Thematic analysis is not connected to a specific ontological or epistemological position; therefore, in this study, the researchers adopted a broadly social constructionist perspective, asserting that all meaning and knowledge are socially created.

L. B. coded all transcripts in NVivo to inductively generate initial codes and co‐researchers coded transcripts from interviews that they had conducted. All codes were copied onto an interactive platform for collaboration (Miro), where they were tentatively grouped according to similar ideas. The full research team met twice to discuss code names and emerging themes, using the miro board as a means of collaboratively working and organizing ideas. All research team members had access to the platform and were free to comment on and edit the arrangement on the Miro board both during and after the meeting. Guided by this input, L. B. revisited the transcripts and codes to iteratively update the codebook. Codes were shared with the co‐researchers for discussion and approval. L. B. and R. W. used this input to develop an initial thematic arrangement, which was iteratively reviewed by the research team members.

Co‐researchers' involvement in analysis was limited by funding restrictions, but researchers sought to maximize the value of their involvement. Co‐researchers were asked which parts of the analysis process they were most interested in doing, and preparatory tasks (e.g., cleaning of transcripts) were completed by L. B. (a full‐time researcher) before sharing transcripts or early codes with the co‐researchers. Naturally, this represents a limitation to the co‐production of knowledge in this project and also reflects the challenges of funding meaningful involvement of young people in research.

## 3 RESULTS

### 3.1 Overall description of themes

Young people had taken part in a wide range of mental health (and broader biopsychosocial) research (Table 1) and this research involvement had taken a variety of forms, including (but not limited to) priority setting, light consultation and youth‐led activities. Experiences discussed took place from approximately 8 years ago up until the present day. Young people had taken part in one or more research projects across the United Kingdom (information on exact geographical location was not gathered).

**Figure 1:**
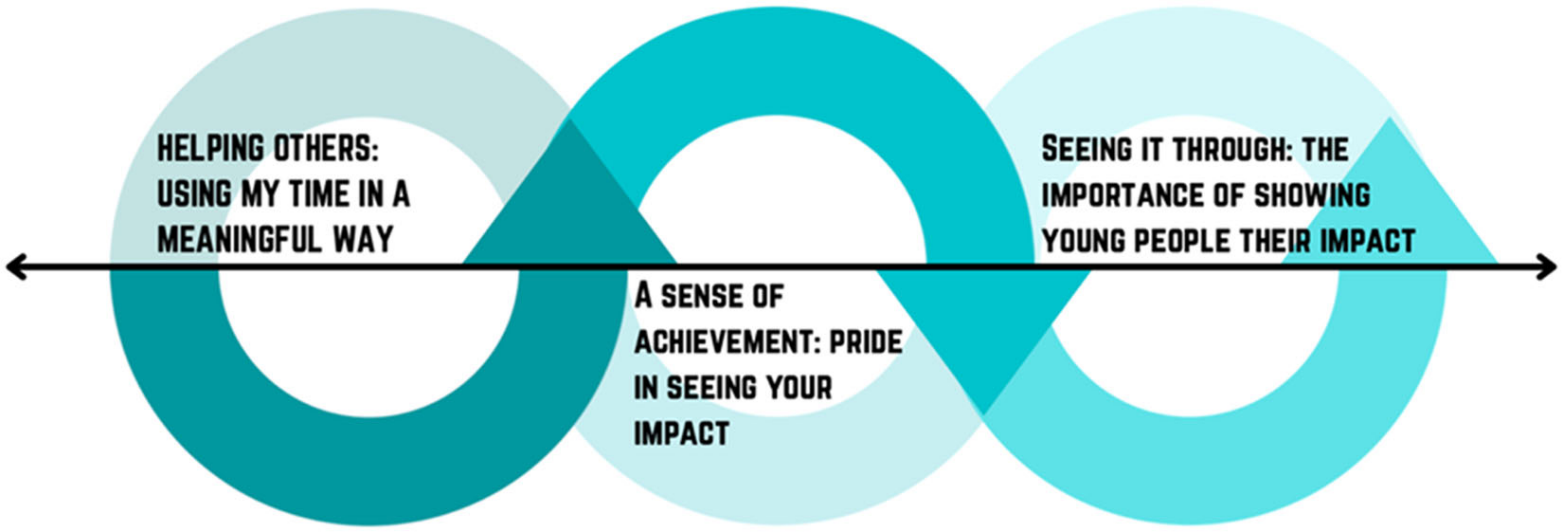
Theme 1: Opportunity to have a meaningful impact. The arrows show the bidirectional relationships between each subtheme—young people want to help out, get involved and gain a sense of pride from their accomplishments, which is facilitated by them seeing the impact that their contributions have had. Being able to see these contributions makes it meaningful and is, therefore, a meaningful use of time, encouraging further involvement and starting the cycle again. The role of the researcher and the mechanism by which it is achieved are not linear, hence the feedback loop represented by the diagram.

**Figure 2:**
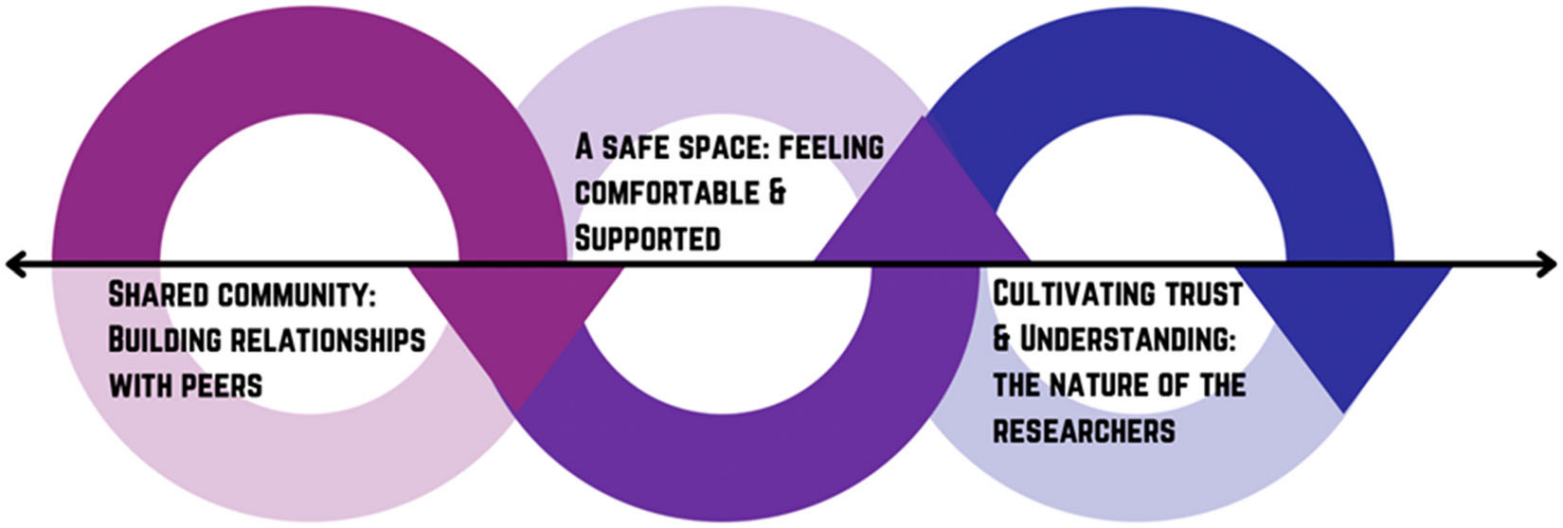
Theme 2: Opportunity to be part of a supportive community. Young people starting to build relationships with one another; these relationships are critical to feeling comfortable and supported in a safe space to share their experiences—without pressure/judgement and knowing that support is available. The influence of the researcher is a critical contributor to their feeling safe and comfortable because they must also be warm, understanding and have respect for the young people who are involved. This, in turn, contributes to the feeling of a shared community because people are able to share their experiences and safely communicate about sensitive topics, further facilitating the ‘safe space’. Again, the subthemes are highly interactive.

**Figure 3:**
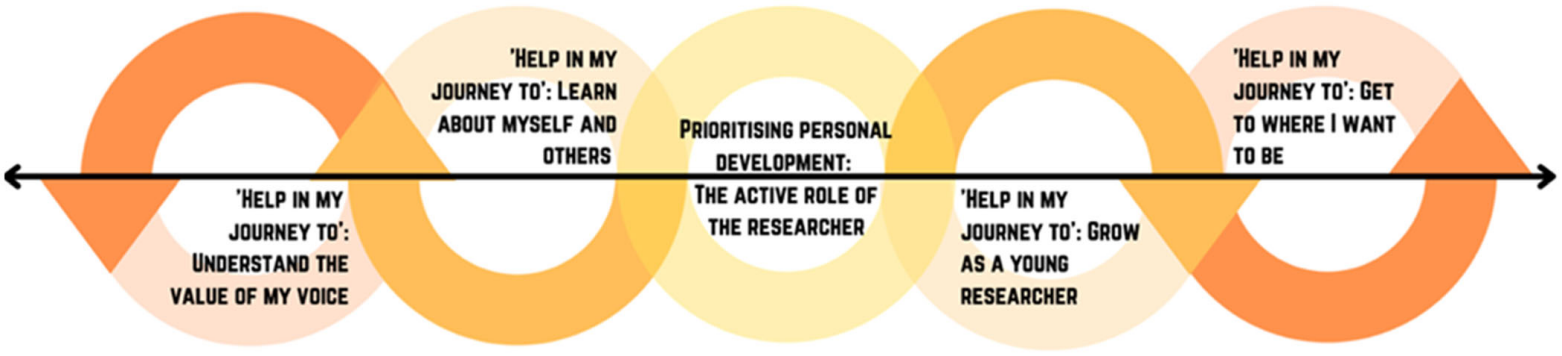
Theme 3: Opportunity to learn and grow. Young people can develop their skills and take away a number of things from their involvement, spanning personal and professional development. Critical to this is the active role of the researcher in investing in young people, by making such development a priority.

**Figure 4:**
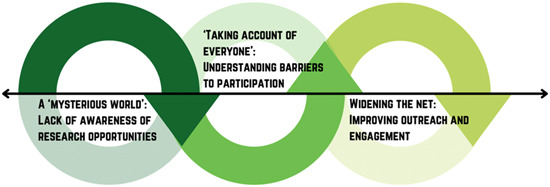
Theme 4: increasing opportunities for young people. This theme maps from problem to solution. While the other themes focus on what young people take away, this theme captures how young people want opportunities to be extended to others so that both young people and research can benefit from their involvement. Research is described as a ‘mysterious world’ and even those who know about research participation might face a number of barriers; this is why it is important to widen the net to reach more young people, which will, in turn, increase awareness. Engagement must then be continually facilitated by understanding the barriers to participation for this wider pool of participants.

**Figure 5:**
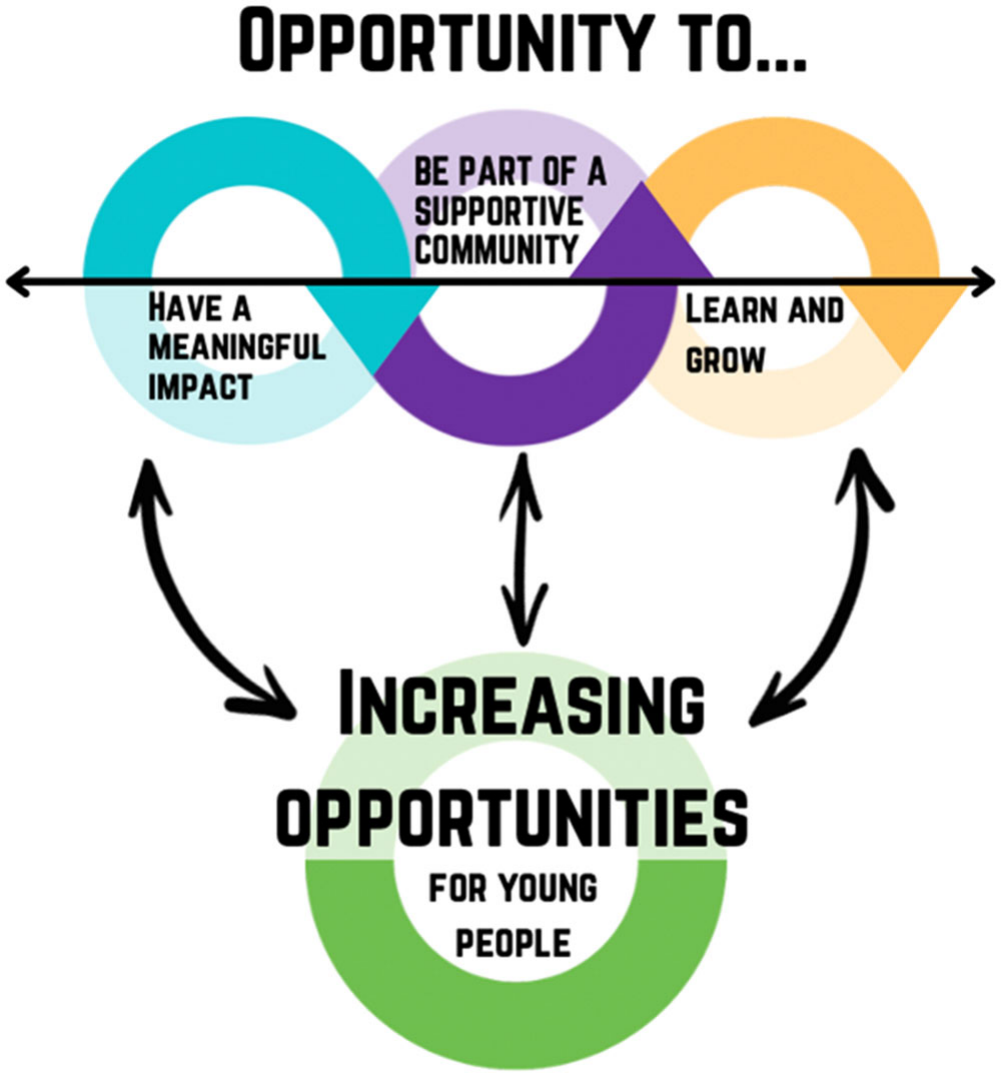
Overall theme diagram: Relationship between themes. Themes 1–3 are about the opportunities that young people are given and what they gain from them, that is, the opportunity to (1) make a difference/have an impact, (2) build relationships/sense of community and (3) to gain skills/personal development. Theme 4 is about how young people find it important that everyone has access to these opportunities, that is, discussing issues around awareness, barriers to participation and ways to reach out more widely to give young people these opportunities.

Overall, experiences were captured in four main themes: (1) opportunity to have a meaningful impact (Figure 1); (2) opportunity to be part of a supportive community (Figure 2); (3) opportunity to learn and grow (Figure 3) and (4) increasing opportunities for young people (Figure 4). Figure 5 shows the relationship between themes.

### 3.2 Theme 1: Opportunity to have a meaningful impact

#### 3.2.1 Subtheme 1. Helping others: Using my time in a meaningful way

Young people described how using their spare time to help improve the lives of others was rewarding and meaningful (‘it felt good to know I was helping other people’, Anya). For many young people, a key motivating factor for being involved in mental health research was knowing that their contribution could have a positive impact on the lives of other young people. Being involved in a research project on a topic that young people had personal experience with, that is, a specific mental health difficulty, gave them the chance to share their unique insights and to listen to and relate to other young people's experiences. It was rewarding for some young people to be able to use their mental health difficulties to create positive change (Table 4—Quote 1).

**Table 4:** Illustrative data for Theme 1.

Number	Name	Quote
Subtheme 1. Helping others: Using my time in a meaningful way		
1	Blake	…another thing which I have enjoyed is being able to relate to other people's experiences and frustrations, both good and bad feelings to some degree, and being able to use that to help shape change and to clearly outline what could be better or what needs improvement and by when and for what purposes.
2	Anya	…it felt good to know that I was helping other people. I'd heard and seen so much about how much other teenagers and other kids were suffering during lockdown, they felt nice to know that, OK, maybe I am actually doing something with my time, not actually just wasting it away sitting here.
3	Julia	…one of the main things was like meeting like people. I feel like meeting like‐minded people that are interested in things like that, like wanting to make positive change and things, were really helpful.
Subtheme 2. A sense of achievement: Pride in seeing your impact		
4	Julia	…with the [research] groups it was like, well, once I've done like one and got like the feeling of like we've accomplished like an actual thing, like we like wrote books, we got like, like new positions in the in Council and things like that.
5	India	I'd probably say … the first project that I got involved with and I guess just because it was a really, it had a lot of impact and we ended up going to the House of House of Commons and we kind of presented it to MPs and things. So that really felt like, you know, we'd achieved something.
Subtheme 3. Seeing it through: The importance of showing young people their impact		
6	Julia	'cause it's so hands on and it was explained to me in a way that made total sense. Like we got to see it from the start to the finish and there was no confusion about what was going on and I feel like the way that I learnt all those things, it's like more beneficial to me now than what I'm learning at uni.
7	Elijah	Well, that's what's been nice with [NAME], she emails…. She emails regularly, and there's that like bit more regular contact, so I think you build up like that a bit of a relationship, don't you, with the person. And then you feel more comfortable. [P] Yeah. Yeah, I do agree with that. Yeah, you do feel more comfortable with like a regular email exchange.
8	Harry	…when they released these little films, when they were finished, when they released them on YouTube, obviously they sent us a link and when I could see like the project doing pretty well in the real world I was I thought that was pretty meaningful and they generally got pretty good responses.

Moreover, young people had the opportunity to meet others with similar experiences and enjoyed working alongside ‘like‐minded people’ (Julia), who were all there to do something impactful with their time (Table 4—Quotes 2–3).

#### 3.2.2 Subtheme 2. A sense of achievement: Pride in seeing your impact

Young people's involvement in research was particularly rewarding when they were able to see evidence of how their involvement had contributed to external outputs (‘we've accomplished like an actual thing’, Julia); for example, Julia and India experienced a strong sense of pride and accomplishment on seeing how their input had contributed to published works and presentations to Members of Parliament. Seeing the impact of their contributions gave young people confidence and a sense of empowerment, realizing that they could actively contribute to something, with limited input from adults, that could help to improve the experiences and lives of others (Table 4—Quotes 4–5).

Involvement in research gave young people an opportunity to share their opinions and knowledge, as well as giving them the chance to show to others that they had more to offer. Young people felt empowered by being able to talk to adults about topics that they were experts on by experience, thus teaching adults things they did not know or might not have considered.

#### 3.2.3 Subtheme 3. Seeing it through: The importance of showing young people their impact

Young people described how being involved in a research project from start to finish made them feel like they had a significant impact on the outcomes of the research project. Making young people's involvement ‘hands on’ (Julia) and including them in decision‐making processes from the very beginning of a project made them feel like they had control over the research and gave them a sense of ownership, which in turn helped their experiences to feel more meaningful (Table 4—Quote 6).

It was important that researchers continued to keep young people updated during and after their involvement to ensure that their input still felt valuable. Participants mentioned that after investing so much time in a project, they liked to keep updated with where the project was at now and to see what their work had achieved over time. Maintaining regular contact with young people was key to sustaining interest and involvement in a project (‘that's what's been nice … she emails regularly’, Elijah). Ensuring that the research team shared all the ‘real world’ (Harry) outcomes of the research with young people was also important, as this showed them how their efforts had contributed towards making a positive impact (Table 4—Quotes 7–8).

### 3.3 Theme 2: Opportunity to be part of a supportive community

#### 3.3.1 Subtheme 1. Shared community: Building relationships with peers

Taking part in research gave participants the opportunity to have ‘fresh interaction[s]’ (Anya) with new people. Several young people who had been involved in in‐person research activities discussed how nonresearch interactions allowed them to form ‘proper bonds’ (Anya) with members of the group and highlighted the importance of building in time and resources for developing friendships between young people. Developing friendships enabled open conversations and progress towards the group's ‘common goal’ (Darren) (Table 5—Quote 1).

**Table 5:** Illustrative data for Theme 2.

Number	Name	Quote
Subtheme 1. Shared community: Building relationships with peers		
1	Anya	every evening we would go out. And it was set in [location], so we would go out and do something. One day we had a pizza making workshop. We went to go see a play or something another day and I think that helped us like make proper bonds with the other people.
2	Blake	…people's lived experiences and perspectives can be so varied, but some things will be similar and in common, and I feel like having that allows people to get to know each other and to be able to relate to one another.
3	India	…so everyone kind of knew and could empathize and understand with you. So it was more of a collaborative environment. It wasn't just kind of like, you know, you're the one with the problem and they're getting it kind of out of you. It's like everyone has a shared understanding.
Subtheme 2. Cultivating trust and understanding: The influence of the researchers		
4	Leah	…that would just kind of like where we started and then you get to know them on a deeper level and in every meeting you'd find out something different about that researcher, you know, in their personal life or their achievements from their past. And that was like an extra connection. Another way to you know, be closer with them and have that bond.
5	India	I think sometimes what I've seen is, you know, researchers get annoyed or angry or just have the expectation that you know this young person will stay on throughout the whole project and attend every meeting and it's like, well, actually that person might not be in the best place mentally or they might not be able to commit to that long period.
Subtheme 3. A safe space: Feeling comfortable and supported		
6	Frankie	It's just people won't understand sometimes outside, but if you're in like a safe environment, I guess, you can talk about it. And then more people will kind of step up it ‘Yeah, I also suffer from that or like I know other people who do’.
7	India	I think they kind of took that as OK well, you don't have any problems speaking about, about it, and we can kind of directly ask you questions about it, and I think perhaps like there wasn't enough communication about my needs at the start and what I was comfortable talking about, because yeah, direct questions were asked and I wasn't always comfortable answering them.

Being involved in research gave participants an opportunity to hear about other young peoples' experiences. Others' experiences were often highly relatable, ‘we really were in the same boat’ (Harry), helping young people to ‘gain a sense of sort of community and support’ (Harry) within the group, based on ‘shared understanding’ (India) (Table 5—Quotes 2–3). However, working online posed a challenge to the formation of these relationships: individuals who had previously taken part in in‐person research noted that online interactions are ‘never going to actually compare’ (Anya) and, in contrast to their in‐person experiences, they had not made friends through online groups, which limited their enjoyment. This was felt less among individuals who had only participated in online research groups, but one young person did note ‘if we were in person then we would probably be quite close to each other. We can bond more’ (Darren).

#### 3.3.2 Subtheme 2. Cultivating trust and understanding: The influence of the researchers

The researchers had a critical influence on the creation of a supportive community. Young people emphasized the importance of researchers coming across like ‘normal people’ (Keira), who were ‘really warm’ and ‘really kind’ (Cara), rather than ‘professors in white lab coats’ (Gemma) who are ‘there just to get information’ (Anya).

Participants highlighted how researchers needed to make a concerted effort to build an initial rapport with young people, for example, by being ‘like 10 times more friendly and approachable than like a normal person’ (Anya) or making time to play games and ‘[having] a bit of a laugh’ (Gemma). Some participants had developed relationships with researchers over longer projects, which allowed them to be more ‘open’ (Gemma) and ‘comfortable’ (Elijah) (Table 5—Quote 4).

A significant aspect of the researcher's role in cultivating a safe space was their acknowledgement, respect and understanding of young people's mental health. The demeanour and behaviour of the researcher had a strong influence on how young people felt in the research environment: being warm, kind and understanding laid the foundation for the development of a supportive community (Table 5—Quote 5).

#### 3.3.3 Subtheme 3. A safe space: Feeling comfortable and supported

Young people described the need to feel comfortable with others in a group to share their experiences. It was essential to cultivate ‘a safe environment’ (Frankie) for young people to talk about sensitive topics that they might not feel able, or have the opportunity, to talk about normally (Table 5—Quote 6). An important part of this was not feeling ‘forced’ or ‘expected to talk about stuff that's happened to [you]’ (Gemma) and that young peoples' choice not to share was accepted by the group. When young people did want to share, it was important that they did not feel like ‘they [were] being judged’ (India) (Table 5—Quote 7).

Emotional support was essential to young people feeling safe to share their views or experiences. It was important that researchers recognized that the subject matter could ‘be quite triggering for people’ (Julia) and that they had enough resources to ‘follow up and check in with young people’ (India). Young people appreciated this ‘reassurance’ (Gemma), which allowed them to share in the knowledge that ‘if they are struggling … there is someone there who can talk with them’ (India). Without this, young people felt that they had to deal with their problems alone.

### 3.4 Theme 3: Opportunity to learn and grow

Theme 3 contains five subthemes. Three subthemes are described here; the remaining two subtheme descriptions can be found in Supporting Information.

#### 3.4.1 Subtheme 1. ‘Help in my journey to’: Grow as a young researcher

Involvement in research provided young people with the opportunity to access practical research experiences and training that they might not learn in other academic settings. Maya spoke about how they applied their experiences to a research methods module because they had ‘actually like [been] a part of research and [seen] how it's done’. One young person spoke about the importance of ‘training certificates’ (India), but these were often not provided.

Numerous young people spoke about how their involvement experiences related to their wider interests and ambitions concerning psychology/research. For several young people, participation in research promoted an interest in psychology‐related studies and careers, both in terms of guiding interests (‘it just helped me know what I kind of wanted to do’, Julia) and providing motivation (‘I started to kind of gain a real interest in psychology, and I thought that's actually what I wanted to do for a career’, India). This interest was often given the chance to grow when young people were involved in multiple projects (Table 6—Quote 1).

**Table 6:** Illustrative data for Theme 3.

Number	Name	Quote
Subtheme 1. ‘Help in my journey to’: Grow as a young researcher		
1	Gemma	I feel like my own interest in research has grown as I've become more involved with it, if that makes sense. I feel like the more I've done, the more I've enjoyed it.
2	Elijah	…it's an inner circle. So like once you're in it, it's like join it so you get opportunities from everywhere else
Subtheme 2. Prioritizing education: The active role of the researcher		
3	Gemma	…gauging like what they know and what they don't know about research so you can try and fill in those gaps. Not assuming that you know stuff when you don't. Like I remember I once came in and it was like, ‘right, so we're doing this randomized controlled trial’ and started talking for like 15 minutes and I was just sat there thinking, what the hell is that, like I've no idea where that is so yeah, not using like jargon or like complicated terms, but making it like friendly for young people without being patronising, which I know is quite fine line but still important.
4	Cara	…they like said it loads of times, ‘do you have any questions?’ And like they didn't make me feel ashamed to like ask something so yeah it was just like really easy for me to ask anything.
Subtheme 3. Learn about myself and others		
5	Julia	…my favorite thing to learn from it is just what other people think, like that is one of my favorite things about research is like, I feel like sometimes when we would be doing analysis and like seeing peoples answers, I'd be like ‘Oh that is totally something that I would think but I didn't even think of that. That's so cool like so so interesting’.
6	Harry	Honestly it was like a whole ‘Yeah, I get what you mean. I get that experience as well’. I think yeah, that was very strong. Again, like I said, we really were in the same boat, so I think just having someone to relate to you, and if you didn't know them that well, you know, even if you'd only met them via screen, it helped to gain a sense of sort of community and support.
7	Maya	I've just become more confident when like talking to people as in like in a group, strangers I've never met before.

Research involvement gave young people access to additional opportunities. Initial participation ‘opened a lot of doors’ (Anya) in terms of research involvement, sometimes giving them access to an ‘inner circle’ (Elijah) of opportunities (Table 6—Quote 2). In addition to finding out about opportunities after involvement, being offered opportunities in between times while they were not working on the current project was ‘really nice’ (Anya).

#### 3.4.2 Subtheme 2. Prioritizing education: The active role of the researcher

Young people described the importance of education, wanting to learn about research as well as more transferable skills. Some participants felt that they were given appropriate training from the start (e.g., India), whereas others felt lost at times (e.g., Gemma). Participants recognized that a sound understanding was important for their own development *and* for the research project so it was worth the investment (Table 6—Quote 3).

Many participants also highlighted the importance of having a supportive environment in which they felt able to ask questions and to clarify their understanding to improve their input. By cultivating an environment in which participants can ask questions when they do not understand, the researchers find out where the gaps in knowledge lie and can then begin to address them (Table 6—Quotes 4). Participants valued the different methods by which training and content were delivered, most notably ‘hands‐on’ learning and interactions (Julia). Similarly, ‘drop‐in sessions’ (India) were highlighted as being useful because participants could come with specific questions to gain a better understanding of the research and/or the project.

Many participants highlighted that they either did like or would have liked researchers to ask them directly what they hoped to gain from their involvement and then act upon those requests. Young people recognized that it was important to reflect upon what they had gained from the project; for example, Frankie shared, ‘it is useful going forward to be able to recognise the skills developed and be able to put them in a personal statement’. Sometimes, this translated into practical support; for example, Elijah was able to make a connection that helped with his GCSE portfolio.

#### 3.4.3 Subtheme 3: ‘Help in my journey to’: Learn about myself and others

Young people experienced personal growth through their involvement in research. For example, Leah described this as ‘a kind of personal journey to understand my mental health better, my gender identity better’.

Young people also expressed that they learned to better understand others' perspectives by talking to other young people about their experiences. For example, Harry described how being involved helped him ‘relate to people … even though, yeah, I'd never really struggled during that time, but I can now understand people who did’. Hearing other people express similar experiences or opinions was comforting and reassuring, whereas hearing different opinions was thought‐provoking and aided learning and growth (Table 6—Quotes 5–6).

Young people also increased in social confidence, which built up over time just by being a part of a group. Several people highlighted that this enabled them to be able to speak in groups and to strangers more confidently (Table 6—Quote 7). Many people shared that they were particularly nervous because it was a new environment, new activities and a lot of new people to meet, but with time, their social anxieties reduced and they felt more comfortable interacting. Further to this, young people also learned to better communicate with others in a group setting. Some received teaching on how to facilitate a group meeting, developing skills to speak with people of different ages and learning practical skills like writing emails.

### 3.5 Theme 4: Increasing opportunities for young people

#### 3.5.1 Subtheme 1. A mysterious world: Lack of awareness of research opportunities

Young people were often unaware that research involvement opportunities existed. For example, young people described how they wanted to get involved, but they ‘didn't know where to start’ or ‘where to look for opportunities’ (Elijah). Even when young people were aware that they could get involved in research, they still had to work hard to search for a particular opportunity.

Young people described that there was an inner circle of involvement whereby young people needed a contact to find out about opportunities. More often than not, young people expressed that they heard about research opportunities from an older relative. For example, ‘my mum works in a university’ (Anya) or ‘I heard about it from my mum who is a psychiatrist’ (Harry), or they heard about the opportunity via ‘a friend of a parent’ (Elijah). It appeared as though this inner circle did not span further than a secondary contact, making research involvement a ‘mysterious world’ (Gemma) that is difficult to access (Table 7—Quotes 1–3).

**Table 7:** Illustrative data for Theme 4.

Number	Name	Quote
Subtheme 1. A mysterious world: Lack of awareness of research opportunities		
1	Gemma	Even now when I try and explain to people what I do in terms of research involvement, they're like—what the hell is that?
2	Anya	…they want to get involved but they don't know where to start or they don't know where to look for these opportunities.
3	Harry	The only reason I know is cause my mum is a psychiatrist who works at [UNIVERSITY] and she has those contacts.
Subtheme 2. ‘Taking account of everyone’: Understanding barriers to participation		
4	Julia	Our lives are like on‐and‐off constantly like sometimes people have school, activities and things like that.
5	Leah	Some of us were an age where we couldn't, couldn't go home on their own because they lived far away, so then coming to the meeting's dependent on whether their parents could pick them up after.
6	Frankie	Sometimes in a household if you not feeling safe talking about what you're going through … it would be harder to be part of this research and all that.
Subtheme 3. Widening the net: Improving outreach and engagement.		
7	Anya	Social media is definitely one of the most powerful tools you could have.
8	Darren	Nearby schools, if you can ask them if you could put up like some posters or inform them in their assemblies or something.
9	Gemma	Especially those from more disadvantaged backgrounds, this is like their window into research and they're never going to see it otherwise because it's quite like a mysterious world unless you've been involved in it.

#### 3.5.2 Subtheme 2. ‘Taking account of everyone’: Understanding barriers to participation

Young people described the importance of researchers respecting and understanding the value of their time. Sometimes, young people felt overcommitted; for example, Anya said ‘it felt like a lot of work because at one point I had like a meeting like, two or three meetings every week’. Young people expressed that involvement was best when it was flexible and when researchers understood when they had other competing priorities.

Working online enabled a unique type of flexibility to make involvement better suited to personal preferences. Young people expressed how they liked that it allowed for anonymity. For example, Gemma said ‘I feel like in person it can be sometimes, it's like more exposing and you feel more vulnerable’.

Research involvement was often easier for young people if they received external support from schools and/or parents. Some young people expressed how this support came from school: for example, ‘having the support of the school and the teachers to give me time off was useful and helpful’ (Julia). Some young people described how taking part depended on support from parents, that is, getting a lift to the sessions, or providing a safe space to talk through difficulties outside of the household (Table 7—Quotes 4–6).

#### 3.5.3 Subtheme 3. Widening the net: Improving outreach and engagement

Based on their own experiences, young people reflected that research is a ‘great’ (Julia) thing to be involved in and discussed how this opportunity should be extended to other young people. One main recommendation from young people was to advertise opportunities in ways that are more accessible to young people. The two main suggestions were via social media (‘social media is one of the most powerful tools you could have’, Anya) and through schools (e.g., ‘you could put up some posters or inform them in their assemblies’, Darren). Young people explained how they spend a lot of spare time on social media, so are more likely to see and engage with content seen there.

Young people also talked about the importance of reaching and involving underrepresented groups. There was particular focus on ethnic groups; young people suggested that researchers should make an active effort to reach out to young people who may not have had the opportunity to have a say: ‘this is their window into research and they're never going to see it otherwise’ (Gemma) (Table 7—Quotes 7–9).

## 4 DISCUSSION: IMPLICATIONS FOR YOUTH MENTAL HEALTH RESEARCHERS

Existing literature tends to focus on the process of involving young people in a particular research project^21^, ^22^ or on problematizing the concept of PPI, either from the perspective of researchers^11^, ^23^, ^24^ or people with lived experience of mental health difficulties.^10^ While this paper touches on some of these topics, it primarily focuses on the benefits for young people who are involved in research between the ages of 11 and 16 years. Findings from this study demonstrate the wide range of such benefits for young people, the ways in which researchers can facilitate these opportunities to maximize the benefits for young people, and the importance of making these opportunities available to all.

Underpinning all of the benefits for young people of being involved in research is the importance of researchers having allocated time and resources to support young people. This includes supporting the development of research skills to improve the quality of the research, but it also includes building supportive relationships, managing expectations and building safe spaces for young people to talk openly and have an open dialogue with the researchers and their peers. Another part of this is managing the expectations of the researchers and ensuring that involvement of young people can be flexible and fit around other commitments of young people. This can be challenging for researchers whose focus is on generating good‐quality research within a traditional academic setting, but it is vital for researchers to consider the needs of young people and the opportunities for providing maximum benefit for young people when they are contributing to academic research. Involvement needs an individualized approach as far as possible, and where not possible, one that leaves room for young people to be able to make genuine choices about how and to what extent they are involved.

The research findings point to several practical actions that researchers can take when involving young people in research to maximize the benefits for the young people of their involvement.

1. Young people say that a key benefit is feeling like they are making a difference and so it is vital that researchers should find ways to show young people the impact that their contribution has had on the research and the impact of the research as a whole. This reflects broader literature about PPI that emphasizes the importance of feeding back to PPI groups about the contribution that they have made to the research.^22^ Honest feedback to young people is important even when there have been (for example) challenges with implementing suggested changes or where funding proposals have been unsuccessful. In addition, involving young people in research outputs and knowledge exchange can support them to feel proud of and empowered by their contributions.
2. It is clear from the findings of this project that young people appreciate being part of a research community in which they feel safe and comfortable. For meaningful involvement to occur, it is essential that researchers are friendly and approachable in their communications with young people, offer times to ‘check in’ with young people individually and have plans in place for supporting those with mental health difficulties as appropriate. Thomson et al.^25^ and McMellon and Mitchell^26^ describe models of young people's involvement in research and PPI, with building a comfortable community and positive relationships at their core. While not every project will have resources to implement this level of activity, young people in this study suggest that building in time for nonresearch interactions and for developing a shared group identity and vision can go some way towards building connections and feeling comfortable.
3. Leading on from the points above, while involvement does not need to be expensive, it is vital that resources are in place to maximize the benefits to young people.^27^, ^28^, ^29^ In funding applications, a sufficient but flexible budget should be costed in to support young people's involvement including, where possible, dedicated time for individuals with the specific skills necessary to support the young people.
4. Finally, taking the benefits to young people of involvement in research seriously requires researchers to make these opportunities for young people to get involved in research accessible, flexible and widely advertised. Young people's perception that existing opportunities are open to a small group of young people with specific skills and existing contacts in academia or ongoing participation in existing youth groups is mirrored in the academic literature (e.g., Brady et al.^27^). It is, therefore, vital that researchers make efforts to ensure that any research opportunity is as accessible as possible, particularly using creative methods to reach underrepresented groups or young people, and advertising opportunities where young people are likely to access them, that is, via schools and social media. Advertising the potential benefits for young people may also help to encourage more young people to get involved in research.

This study highlights young people's experiences of being involved in mental health research across multiple projects and identifies ways in which researchers can incorporate these findings into their future projects to help maximize the benefits not just to the research but also to young people. These benefits cannot be separated from the benefits to the research of involving young people, and we argue that working with co‐researchers in this research has both improved the research and benefited the co‐researchers.

## AUTHOR CONTRIBUTIONS

Rebecca Watson and Cathy Creswell led on all aspects of the project. Lowrie Burgess, Elise Sellars, Christina McMellon and Rachel Temple contributed to study design, supported data collection and contributed to the analysis. Jodie Crooks, Rose McGowan, James Diffey, Georgia Naughton, Rebekah Carrington and Cassie Lovelock were peer researchers who contributed to study design, data collection and analysis. All authors contributed to the study write‐up.

## CONFLICT OF INTEREST STATEMENT

The authors declare no conflict of interest.

## ETHICS STATEMENT

Ethical approval for the study was obtained from the University of Oxford Medical Science Division Research Ethics Committee. Participants older than 16 years of age and parents of participants younger than 16 years of age gave their verbal consent; participants younger than 16 of age also provided their assent.

## Supporting information

Supporting information.

## Data Availability

The data that support the findings of this study are available on request from the corresponding author. The data are not publicly available due to privacy or ethical restrictions.
