# Supplementary material for: A qualitative study exploring the benefits of involving young people in mental health research
Source: Health Expect. 2023 Apr 19;26(4):1491–504. doi: 10.1111/hex.13722 (PMC10349260; doi:10.1111/hex.13722)
Supplement: Supplementary file 1 — Supporting information. [file HEX-26--s001.docx]

**Supplementary Material**

Topic Guide

Codes, Sub-Themes, Themes

Theme 3. Sub-theme. ‘Help in my journey to’: Understand the value of my voice

Theme 3. Sub-theme. ‘Help in my journey to’: Get where I want to be

**Topic Guide**

**OVERALL RESEARCH QUESTION. Exploring young people’s experiences of being involved in mental health research]** Tell me about your experience(s) of being involved in mental health research…

**RESEARCH QUESTION 1. UNDERSTANDING MORE ABOUT THE WAYS YOUNG PEOPLE ARE INVOLVED IN MENTAL HEALTH RESEARCH]** In your expression of interest form, you mentioned that you had taken part in [insert answer(s)] research, can you tell me a bit more detail about these experiences?

(Specific prompts to use if helpful/relevant)

- How long did you do these projects for?
- Did you take part alone or with a small group?
- What made you want to take part in this research?
- What did [insert answer] involve you doing?
- Was there anything you liked or enjoyed about this?
- Was there anything you found challenging or difficult about this?
- **Face to face vs. Online** Have you taken part in any research virtually since March 2020? If so, how have you found this is comparison to in person research? [e.g. anything you have preferred, anything you have not liked as much?]

**RESEARCH QUESTION 2. UNDERSTANDING WHAT MAKES INVOLVEMENT IN RESEARCH ENJOYABLE/MEANINGFUL/USEFUL FOR YOUNG PEOPLE]** Can you tell me more about what made these experiences enjoyable or meaningful or useful for you (e.g. felt like taking part was worthwhile for you)?

(Specific prompts to use if helpful/relevant)

- What did you enjoy the most?
- What specifically made X experience enjoyable or meaningful or useful? *[e.g. If someone enjoyed working in a group to think about research projects, was this because they like working collaboratively/learning about other people’s views/ they made new friends etc.?]*
- What did the researchers do that helped make X enjoyable or meaningful or useful?
- Is there anything you learnt/gained from the more challenging or difficult experiences? *[i.e. even though it was difficult, did you gain a skill/insight about yourself- e.g. if someone found speaking in a large group difficult, they gained confidence in public speaking]*
- Were there any ways that the researchers helped you get these benefits?
- What could have made X challenging/difficult experience more enjoyable/useful/meaningful? *[i.e. how could the experience have been improved?]*
- *[If taken part in multiple projects/involvement opportunities]:*
- Thinking about the different projects you have been involved in, which type of involvement do you feel you have enjoyed/benefitted the most from and why?
- What helped that to happen?
- Do you feel the different projects have they have built on each other/ supplemented each other in ways that have worked well for you *[e.g. I learnt X in the first one then developed X in the second one]*
- Which type of involvement do you feel you have enjoyed/benefitted the least from and why?
- What might have helped that be more enjoyable or useful?
- Did your experiences of taking part match your expectations of when you signed up? If not, why?

**RESEARCH QUESTION 3. UNDERSTANDING WHAT SKILLS/KNOWLEDGE/PERSONAL DEVELOPMENT YOUNG PEOPLE HAVE GAINED FOR BEING A PART OF MENTAL HEALTH RESEARCH.** Are there any skills or knowledge or other benefits you feel you’ve gained or developed from taking part in research?

- What did you learn? *[can be general- e.g. knowledge about an area of research- what was the project about? Or more specific- e.g. particular research skills, such as data analysis]*
- What would you have liked to learn about? *[ie. A type of involvement (e.g. youth-led research) or area of research more generally (e.g. mental health research)]*
- Did you have the opportunity to discuss your goals/ ambitions for skill development with researchers at the start and reflect with them at the end of the project? If so, how did you find this? If not, would you have liked to?
- Did being involved in research have any wider benefits for you in your life? *[e.g. opening up conversations about mental health with family or friends? Inform interest in a career in psychology/research?]*
- [If skills learnt] How have you used this skill(s) in other parts of your life? *[e.g. if you feel you gained confidence public speaking, have you used this at school/ in any clubs you are part of?]*
- THEN [or if no skills gained]
- What skills would you have liked the opportunity to develop and why? *[can think about this in terms of personal skills (e.g. confidence/friendship) and research skills (e.g. data analysis)]*
- Are there other ways that you think taking part in mental health research has benefitted you or the people around you? Or other ways that you would have liked it to have brought benefits for you and/or the people around you?

**RESEARCH QUESTION 4. UNDERSTANDING HOW YOUNG PEOPLE THINK THEY SHOULD BE INVOLVED IN MENTAL HEALTH RESEARCH]** Thinking a bit more generally, are there any other types of research involvement opportunities you think young people should be more involved in/ you would like to be involved in [and why?]

(Specific prompts to use if helpful/relevant)

- What kind of research opportunities do you think should be available for young people to develop research skills/ confidence etc.? *[e.g. more opportunities for youth-led research, so that young people can develop their independent research skills]*
- *OR* If you were going to take part in another project, what would you want to focus on/learn/develop?
- *OR* What would your ideal research project look like for you and why?
- How/why do you think young people would benefit from these experiences? *[i.e. what skills/experiences do you think young people would gain? And why do you think that is important?]*
- What would you like to be able to give to research and why? – *[i.e. some might have a goal of being able to make positive contributions as well as just developing their own skills?]*

**RESEARCH QUESTION 5. UNDERSTANDING HOW RESEARCHERS CAN BETTER SUPPORT YOUNG PEOPLE’S INVOLVEMENT IN MENTAL HEALTH RESEARCH]** Again, thinking a bit more generally, in the future, how do you think researchers could better support young people to be involved in research in ways which make this a positive experience for young people?

(Specific prompts to use if helpful/relevant)

Based on your research experience…

- Are there any particularly important messages you’d like to share with researchers about the best ways to involve young people in mental health research? Both to make sure there are benefits for the research and to the young people themselves
- Are there any messages you’d like to share about what ‘not to do’/ stop doing when involving young people in research?
- Is there anything you think researchers could keep doing or start doing in the future to help young people develop skills/experience/get other benefits? *[e.g. types of training/ workshops focusing on particular skills? Types of involvement opportunities that you think young people gain the most skills/experience from?]*
- Is there anything you think researchers could do better in the future to help young people find involvement in research enjoyable/meaningful? *[e.g thinking about the whole research process- from signing up for research, consent forms etc. Also ways to ensure young people feel their voice is heard/ they have played an active role in the research? Ways to make sessions more interactive etc.]*

**Codes, Sub-Themes, Themes**

| **Themes** | **Sub-themes** | **Codes** |
| --- | --- | --- |
| THEME 1. Opportunity to have a meaningful impact | SUB-THEME 1. Helping others: Using my time in a meaningful way | *‘It felt good to know I was helping other people’ - having the desire to affect positive change* |
|  |  | *‘Having something to do’ - using my free time to ‘help out’* |
|  | SUB-THEME 2. A sense of achievement: Pride in seeing your impact | *A sense of “reward”: ’Knowing that I’d actually made a difference’* |
|  |  | *Feeling ‘proud’ of your accomplishments* |
|  |  | *Feeling empowered - showing adults what young people have to offer* |
|  | SUB-THEME 3. Seeing it through: The importance of showing young people their impact | *‘It resulted in a bunch of content that I actually think is useful’ -seeing positive outcomes makes it meaningful* |
|  |  | *‘I felt like I was involved all the way throughout’ - the significance of seeing it from start to finish* |
|  |  | *Wanting to be kept in the loop during a project and beyond – ‘keeping us updated with what we’re helping to’* |
| THEME 2. Opportunity to be part of a supportive community | SUB-THEME 1. Shared community: Building relationships with peers | *‘Proper bonds’ - building meaningful relationships with peers* |
|  |  | *The importance/value of building in time for non-research interactions with peers* |
|  |  | *Having the chance to meet new people and make friends* |
|  |  | *Relating to others through shared experiences - ‘being in the same boat’* |
|  |  | *Having a common goal: collaborating with others who ‘genuinely wanted to make a difference’* |
|  |  | *Generating ideas - having people to ‘bounce off of’* |
|  |  | *Online engagement shifts the (relationship) dynamics - ‘It can come close but it’s never going to compare’* |
|  |  | *Struggling to bridge the age gap – feeling ‘daunted’ by the differences* |
|  | SUB-THEME 2. A safe space: Feeling comfortable and supported | *Being open and honest - having a safe space to talk about issues* |
|  |  | *Getting ‘comfortable’ in a group – experiencing a ‘community’ feel* |
|  |  | *‘Tell us as much as you want’ – having no ‘pressure’ to contribute* |
|  |  | *‘Duty of care’ – feeling emotionally supported when discussing sensitive topics* |
|  | SUB-THEME 3. Cultivating trust and understanding: The influence of the researcher | *‘Building rapport’ – being listened to and treated ‘as a person’ by the researchers* |
|  |  | *Valuing ‘continuity’ –building relationships with the researcher over time* |
|  |  | *‘Everyone is at a different stage in their journey’ – understanding the potential impact of involvement on personal mental health* |
|  |  | *Balancing time commitments – respecting and valuing our / young people’s time and resources* |
| THEME 3. Opportunity to learn and grow: ‘Help in my journey to…’ | SUB-THEME 1. ‘Help in my journey to’: Learn about myself and others | *Developing my emotional literacy* |
|  |  | *Gaining social confidence* |
|  |  | *Learning to communicate with others* |
|  |  | *Learning about other perspectives* |
|  |  | *Overcoming initial social anxiety* |
|  | SUB-THEME 2. ‘Help in my journey to’: Understand the value of my voice | *Gaining confidence in my voice and skillset* |
|  |  | *Feeling like my voice is valued*  *Being viewed as ‘a whole person’* |
|  |  | *‘Young people know what is best for young people’ – having the opportunity to give my unique input* |
|  | SUB-THEME 3. ‘Help in my journey to’: Grow as a young researcher | *‘It made me more aware of the issues’ – having the chance to learn more about mental health* |
|  |  | *Having access to practical research experience and training* |
|  |  | *Promoting further interest in psychology (careers)* |
|  |  | *Gaining access to additional opportunities* |
|  | SUB-THEME 4. ‘Help in my journey to’: Get to where I want to be | *Gaining experiences for my CV* |
|  |  | *Skilling up for my academic and professional career* |
|  |  | *Tangible recognition – providing certification/awards* |
|  |  | *Financial incentives – “I’m helping other people and also getting something out of it for myself”* |
|  | SUB-THEME 5. Prioritising personal development: The researcher as an active facilitator | *Prioritising education: ensuring learning and training are supported by researchers* |
|  |  | *Choice and autonomy – asking young people what they want to take away* |
|  |  | *Researchers make involvement enjoyable* |
|  |  | *Setting expectations - young people want clarity about their participation* |
| THEME 4. Increasing opportunities: ‘Everyone should get the opportunity to be part of a research program’ | SUB-THEME 1. A ‘mysterious world’: Lack of awareness of research opportunities | *Lack of awareness: “I didn’t even know it existed”* |
|  |  | *The inner circle: Involvement via personal connections* |
|  | SUB-THEME 2. ‘Taking account of everyone’: Understanding barriers to participation | *Respecting and understanding young people’s other commitments/constraints* |
|  |  | *Importance of external support, e.g., school, parents* |
|  |  | *Working online enables unique flexibility to personal preferences* |
|  | SUB-THEME 3. Widening the net: Improving outreach and engagement | *Advertising through schools and social media* |
|  |  | *Importance of reaching underrepresented groups* |
|  |  | *Online opportunities increase accessibility* |

**Theme 3. Sub-theme. ‘Help in my journey to’: Understand the value of my voice**

By sharing their opinions and ideas, young people were able to learn about the value of their contributions. Participants highlighted that they could be hesitant to contribute, or lack confidence in the value of their opinion (“Oh my God, why am I here?” [Gemma]), but that their involvement allowed them to gain confidence in their voice as a young person. This wasn’t necessarily taught: young people gained confidence just by taking part in activities and events.

An important facet of this learning experience was the response of the researchers. It was important that young people felt heard and understood, and that researchers “really wanted to listen to us” [Anya] – which was actively demonstrated by researchers writing things down and “following on from our points, not just like sort of “OK thanks, next”” [Harry]. “Positive feedback” [Julia] provided reassurance, as did seeing how their “viewpoints shaped projects” [Gemma], offering young people a learning opportunity to realise “Oh my opinion is important” [Gemma]. Positive feedback and active listening meant that their involvement didn’t feel “tokenistic” [Leah]: it was important that young people were viewed as “a whole person, not there just to kind of give you a PPI perspective" [India].

Following on from this discussion about the value of young people’s input, participants strongly advocated for youth-led research (“anything that young people can be involved in, they should be” [Anya]). Participants had a keen understanding of how “young people know what is best for young people” [Julia] and highlighted the need to centralize young people’s voices throughout the research process.

**Theme 3. Sub-theme. ‘Help in my journey to’: Get where I want to be**

Participation in mental health research gave young people a range of transferable skills and experiences that they could utilise in their academic and/or professional careers; for example, many improved their knowledge of psychology, gained leadership skills and confidence, and, overall, enhanced their resumes (“I had so much to write on my CV” [India]). However, participants highlighted that they were not always aware of the skills they gained or how these might transfer to other parts of their lives, and that it might be useful for researchers to dedicate some time to explaining this to young people (“if someone from kind of the research team had sat and talked through certain things that I might have had, like a better understanding of it” [India]).

Building on this, participants expressed that tangible recognition – in the form of certificates or awards - would be useful. Financial incentives were seen as an added bonus but were generally not the impetus behind young people’s participation (“it's nice to help out and had a lot of free time… then someone told me I was getting paid and I was like “oh OK that's even better”” [Elijah]).
